# Commentary: When patient severity signals family vulnerability – reframing ICU family needs as a nursing priority

**DOI:** 10.1177/17449871261466747

**Published:** 2026-08-31

**Authors:** Abdishakur Mohamud Hassan Hidigow

**Affiliations:** Senior ICU Nurse, Department of Intensive Care Unit, Dr. Sumait Hospital, SIMAD University, Mogadishu, Somalia Department of Nursing and Midwifery, Faculty of Medicine and Health Sciences, SIMAD University, Mogadishu, Somalia

**Commentary to:** Exploring the association between family needs and clinical status in intensive care unit patients: a cross-sectional study (Regalado et al., 2026).

Admission to an intensive care unit (ICU) is rarely experienced by families as a single clinical event. It is often a sudden disruption of normal family life, marked by uncertainty, fear, unfamiliar technology and urgent dependence on healthcare professionals for information. For nurses, this means that critical care is never limited to physiological stabilisation alone. The patient’s family becomes part of the field of care, especially when the patient is unconscious, sedated, mechanically ventilated or unable to communicate. In this context, the paper by Regalado et al. (2026) offers a timely and clinically meaningful contribution by examining how the clinical status of critically ill patients is associated with the perceived needs of their next of kin.

Regalado et al. (2026) reports a cross-sectional dyadic study conducted in a Portuguese ICU involving 140 critically ill patient–next-of-kin dyads. The study examined whether objective indicators of patient clinical status were associated with the intensity of family needs. Patient status was assessed using established tools, including the Coma Recovery Assessment Instrument of the University of Aveiro, the Glasgow Coma Scale, the Richmond Agitation-Sedation Scale, the Numeric Pain Scale and the Behavioural Pain Scale. Family needs were assessed using the Family Needs Inventory in the ICU. The paper’s main message is clear and highly relevant to nursing: families’ needs are not static during critical illness. Rather, they appear to intensify when the patient’s clinical and neurobehavioural condition is poorer.

The study found that next of kin placed high importance on needs related to participation in care and information. This is unsurprising to many ICU nurses, but the study strengthens this everyday clinical observation by linking family needs with measurable clinical indicators. Poorer scores on consciousness and neurobehavioural assessment tools, deeper sedation and invasive mechanical ventilation were associated with greater perceived family needs. Female relatives, ventilatory support and relationship to the patient were also identified as independent predictors of need intensity. These findings are important because they help move family-centred care from a general principle to a more targeted nursing response.

From a nursing theory perspective, the paper supports patient- and family-centred care by showing that the family’s distress and informational needs are closely connected to the patient’s clinical condition. This aligns with the wider literature, which recognises families as partners in intensive care and emphasises communication, respect, participation and shared decision-making as essential components of high-quality care (Davidson et al., 2017; Schwartz et al., 2022). It also resonates with family systems thinking: when one member of the family is critically ill, the whole family system is affected. A sedated or ventilated patient does not only represent increased physiological instability; for relatives, this may also represent silence, uncertainty and fear of deterioration or death.

As a senior ICU nurse, the findings reflect a familiar pattern in clinical practice. Families often become most anxious when the patient cannot speak, respond or show visible signs of improvement. A ventilator, infusion pumps, sedation and reduced consciousness may be routine clinical realities for ICU staff, but to relatives they can signal danger, helplessness and loss of control. In such situations, the family’s repeated questions are not merely requests for information; they are attempts to restore meaning and emotional stability. This is why therapeutic communication is not an optional soft skill in critical care nursing. It is a core safety and quality intervention.

The practical implications are wide. Bedside ICU nurses can use changes in consciousness, sedation level and ventilatory support as prompts to assess family vulnerability more actively. For example, a deeply sedated or invasively ventilated patient should trigger not only closer physiological monitoring but also more proactive family communication. Nurse educators can use this study to teach students that family assessment is part of critical care assessment, not an additional task performed only when time permits. ICU managers can use the findings to justify structured family information pathways, family liaison roles, communication training and policies that support appropriate family participation in care.

The paper is also relevant for nursing policy. If family-centred care is to be taken seriously, it needs to be embedded into ICU quality indicators, documentation systems and staff development programmes. Structured communication protocols, scheduled family updates, clear explanation of clinical equipment and flexible visiting arrangements where clinically appropriate may help reduce uncertainty and support family involvement. These measures are especially important because family distress after ICU admission can contribute to post-intensive care syndrome–family, including anxiety, depression and post-traumatic stress symptoms (Halain et al., 2022; Leong et al., 2023). The paper therefore reminds us that preventing harm in ICU includes attention to psychological and relational harm experienced by families.

Although the study was conducted in Portugal, its message has wider international relevance. In resource-limited ICUs, including settings where staffing shortages, overcrowding, restricted visiting systems and limited communication training are common, family needs may be even more difficult to identify and address. Nurses in such environments often work under intense pressure, yet they remain the professionals most continuously present at the bedside. This makes nursing leadership essential in developing realistic, low-cost family-centred interventions, such as consistent family updates, simple explanations of patient status, culturally sensitive communication and early identification of relatives who may be at higher emotional risk.

The strength of Regalado et al. (2026) lies in making visible a relationship that ICU nurses often sense but may not formally document: when the patient is clinically more vulnerable, the family may also be more vulnerable. The paper encourages nurses to read clinical severity not only as a patient-monitoring issue but also as a family-care signal. This reframing is valuable for practice, education, management and policy. Ultimately, the paper reminds us that family needs are not secondary to ICU care. They are closely connected to patient condition, communication, safety and recovery, and they deserve a deliberate place in the work of critical care nursing.

## References

[bibr1-17449871261466747] Davidson JE Aslakson RA Long AC , et al. (2017) Guidelines for family-centered care in the neonatal, pediatric, and adult ICU. Critical Care Medicine 45: 103–128. 27984278 10.1097/CCM.0000000000002169

[bibr2-17449871261466747] Halain AA Tang LY Chong MC , et al. (2022) Psychological distress among the family members of intensive care unit patients: A scoping review. Journal of Clinical Nursing 31: 497–507. 34254377 10.1111/jocn.15962

[bibr3-17449871261466747] Leong E Chew CC Ang J , et al. (2023) The needs and experiences of critically ill patients and family members in intensive care unit of a tertiary hospital in Malaysia: A qualitative study. BMC Health Services Research 23: 966. 37312146 10.1186/s12913-023-09660-9 PMC10262139

[bibr4-17449871261466747] Regalado BC Alvarelhão JJM Lindo Simões JFF (2026) Exploring the association between family needs and clinical status in intensive care unit patients: a cross-sectional study. Journal of Research in Nursing. Epub ahead of print 23 August 2026. DOI: 10.1177/17449871261456573. 10.1177/17449871261456573 PMC13503640 42643491

[bibr5-17449871261466747] Schwartz A Dunn SL Simon H , et al. (2022) Making family-centered care for adults in the ICU a reality. Frontiers in Psychiatry 13: 837708. 35401268 10.3389/fpsyt.2022.837708 PMC8987300

